# 2-Fluoro-*N*-*o*-tolyl­benzamide

**DOI:** 10.1107/S1600536808043122

**Published:** 2008-12-24

**Authors:** Aamer Saeed, Rasheed Ahmad Khera, Shahid Ameen, Jim Simpson, Roderick G. Stanley

**Affiliations:** a Department of Chemistry, Quaid-i-Azam University, Islamabad 45320, Pakistan; b Department of Chemistry, University of Otago, PO Box 56, Dunedin, New Zealand

## Abstract

In the title compound, C_14_H_12_FNO, the *ortho*-F atom and corresponding H atom on the fluoro­benzene ring are disordered over two positions with occupancies of 0.856 (4) and 0.144 (4). The amide unit is planar with a maximum deviation of 0.0057 (16) Å and the amide plane makes dihedral angles of 38.27 (11)° with the fluoro­benzene ring plane and 37.53 (10)° with the tolyl ring. The two benzene rings are inclined at an angle of 4.17 (15)°. In the crystal structure, chains form along *b* through N—H⋯O hydrogen bonds augmented by C—H⋯π inter­actions. Additional inter­molecular C—H⋯O and C—H⋯F hydrogen bonds further stabilize the structure, forming layers in the *ac* plane.

## Related literature

For related structures, see: Chopra & Guru Row (2008 ▶); Donnelly *et al.* (2008 ▶); Hou *et al.* (2004 ▶); Saeed *et al.* (2008 ▶). For reference structural data, see: Allen *et al.* (1987 ▶).
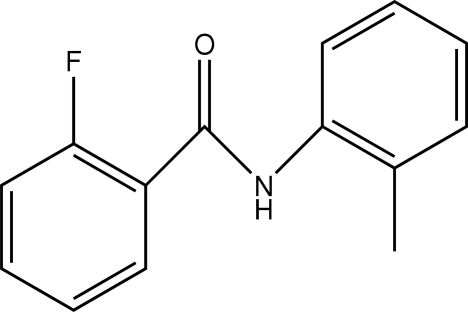


## Experimental

### 

#### Crystal data

C_14_H_12_FNO
*M* *_r_* = 229.25
Monoclinic, 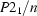

*a* = 10.749 (4) Å
*b* = 4.8245 (17) Å
*c* = 21.580 (7) Å
β = 93.820 (19)°
*V* = 1116.6 (7) Å^3^
*Z* = 4
Mo *K*α radiation
μ = 0.10 mm^−1^
*T* = 93 (2) K
0.25 × 0.15 × 0.05 mm

#### Data collection

Bruker APEXII CCD diffractometer
Absorption correction: multi-scan (*SADABS*; Bruker, 2006 ▶) *T*_min_ = 0.779, *T*_max_ = 0.995
7896 measured reflections
1228 independent reflections
925 reflections with *I* > 2σ(*I*)
*R*_int_ = 0.067
θ_max_ = 21.3°

#### Refinement

*R*[*F* ^2^ > 2σ(*F* ^2^)] = 0.037
*wR*(*F* ^2^) = 0.108
*S* = 1.06
1228 reflections
169 parameters
H atoms treated by a mixture of independent and constrained refinement
Δρ_max_ = 0.19 e Å^−3^
Δρ_min_ = −0.21 e Å^−3^

### 

Data collection: *APEX2* (Bruker 2006 ▶); cell refinement: *APEX2* and *SAINT* (Bruker 2006 ▶); data reduction: *SAINT*; program(s) used to solve structure: *SHELXS97* (Sheldrick, 2008 ▶); program(s) used to refine structure: *SHELXL97* (Sheldrick, 2008 ▶) and *TITAN2000* (Hunter & Simpson, 1999 ▶); molecular graphics: *SHELXTL* (Sheldrick, 2008 ▶) and *Mercury* (Macrae *et al.*, 2006 ▶); software used to prepare material for publication: *SHELXL97*, *enCIFer* (Allen *et al.*, 2004 ▶), *PLATON* (Spek, 2003 ▶) and *publCIF* (Westrip, 2009 ▶).

## Supplementary Material

Crystal structure: contains datablocks I. DOI: 10.1107/S1600536808043122/hb2882sup1.cif

Structure factors: contains datablocks I. DOI: 10.1107/S1600536808043122/hb2882Isup2.hkl

Additional supplementary materials:  crystallographic information; 3D view; checkCIF report

## Figures and Tables

**Table 1:** Hydrogen-bond geometry (Å, °)

*D*—H⋯*A*	*D*—H	H⋯*A*	*D*⋯*A*	*D*—H⋯*A*
N1—H*N*1⋯O1^i^	0.85 (3)	2.08 (3)	2.887 (3)	158 (2)
C6—H6⋯F1′^ii^	0.95	2.60	3.219 (11)	123
C12—H12⋯O1^iii^	0.95	2.63	3.434 (3)	143
C12—H12⋯F1′^iii^	0.95	2.40	3.287 (11)	154
C14—H14*C*⋯*Cg*1^i^	0.98	2.98	3.702 (3)	131

Symmetry codes: (i) 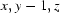; (ii) 
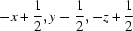
; (iii) 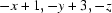. *Cg*1 is the centroid of the C8–C13 benzene ring.

**Fig. 1.**
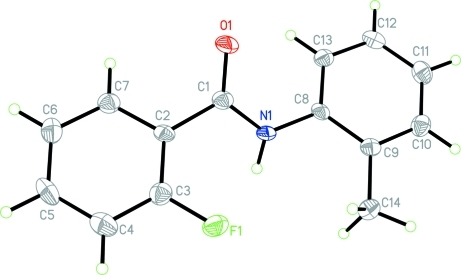
The molecular structure of (I) with displacement ellipsoids for the non-hydrogen atoms drawn at the 50% probability level. For clarity atoms of the minor disorder component have been omitted.

**Fig. 2.**
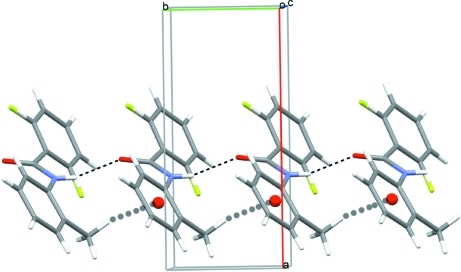
Chains formed along a by N—H···O hydrogen bonds (dashed lines) and C—H···π interactions (dotted lines). Spheres represent the centroids of C8···C13 benzene rings.

**Fig. 3.**
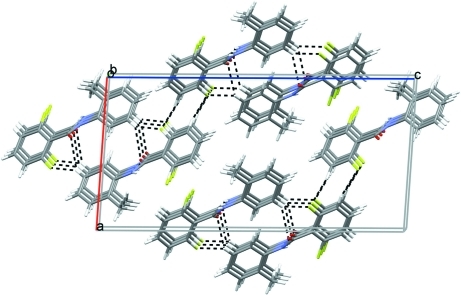
Crystal packing of (I) viewed down the b axis with hydrogen bonds drawn as dashed lines.

## supplementary crystallographic information

### Comment

As part of our ongoing work on the structure of benzanilides and related compounds (Saeed *et al.*, 2008), we report here the structure of the title 2-fluorobenzamide derivative, (I), Fig 1. The C2—C1—O1—N1—C8 unit is almost planar with a maximum deviation of 0.0057 (16)Å for N1. This plane makes dihedral angles of 38.27 (11) ° with the fluorobenzene ring plane and 37.53 (10)° with the tolyl ring. The two benzene rings are inclined at an angle of 4.17 (15)° giving the molecule a stepped structure. Bond distances in the molecule are not unusual (Allen *et al.*, 1987) and agree well with those reported previously (see for example Chopra & Guru Row, 2008; Donnelly *et al.*, 2008; Hou *et al.*, 2004, Saeed *et al.*, 2008).

In the crystal structure, chains form along *b* through N—H···O hydrogen bonds augmented by C—H···π interactions involving the methyl group and the tolyl rings (Table 1, Fig. 2). Additional intermolecular C—H···O and C—H···F hydrogen bonds further stabilize the structure forming layers in the *ac* plane, that stack down the *b* axis (Fig. 3).

### Experimental

2-Fluorobenzoyl chloride (5.4 mmol) in CHCl_3_ was treated with 2-methylaniline (21.6 mmol) under a nitrogen atmosphere at reflux for 3 h. Upon cooling, the reaction mixture was diluted with CHCl_3_ and washed consecutively with 1 *M* aqueous HCl and saturated aq NaHCO_3_. The organic layer was dried over anhydrous magnesium sulfate and concentrated under reduced pressure. Crystallization of the residue from CHCl_3_ afforded (I) as colourless needles in an81% yield: Anal. calcd. for C_14_H_12_FNO: C 73.35, H 5.28, N 6.11%; found: C 73.30, H 5.32, N 6.09%

### Refinement

The H atom bound to N1 was located in a difference electron density map and refined freely with an isotropic displacement parameter. All other H-atoms were refined using a riding model with C—H = 0.95 Å, *U*_iso_= 1.2*U*_eq_ (C) for aromatic and 0.98 Å, *U*_iso_ = 1.5*U*_eq_ (C) for methyl H atoms. The *ortho*-F atom and corresponding H atom on the fluorobenzene ring are disordered over two positions with occupancies 0.856 (4) and 0.144 (4). Crystals were very small and weakly diffracting, with no significant data obtained beyond θ = 21°

### Crystal data

**Table:**

C_14_H_12_FNO	*F*(000) = 480
*M**_r_* = 229.25	*D*_x_ = 1.364 Mg m^−^^3^
Monoclinic, *P*2_1_/*n*	Mo *K*α radiation, λ = 0.71073 Å
Hall symbol: -P 2yn	Cell parameters from 1374 reflections
*a* = 10.749 (4) Å	θ = 3.3–21.2°
*b* = 4.8245 (17) Å	µ = 0.10 mm^−^^1^
*c* = 21.580 (7) Å	*T* = 93 K
β = 93.820 (19)°	Rectangular plate, colourless
*V* = 1116.6 (7) Å^3^	0.25 × 0.15 × 0.05 mm
*Z* = 4	

### Data collection

**Table:**

Bruker APEXII CCD diffractometer	1228 independent reflections
Radiation source: fine-focus sealed tube	925 reflections with *I* > 2σ(*I*)
graphite	*R*_int_ = 0.067
ω scans	θ_max_ = 21.3°, θ_min_ = 1.9°
Absorption correction: multi-scan (*SADABS*; Bruker, 2006)	*h* = −10→10
*T*_min_ = 0.779, *T*_max_ = 0.995	*k* = −4→4
7896 measured reflections	*l* = −22→22

### Refinement

**Table:**

Refinement on *F*^2^	Primary atom site location: structure-invariant direct methods
Least-squares matrix: full	Secondary atom site location: difference Fourier map
*R*[*F*^2^ > 2σ(*F*^2^)] = 0.037	Hydrogen site location: inferred from neighbouring sites
*wR*(*F*^2^) = 0.108	H atoms treated by a mixture of independent and constrained refinement
*S* = 1.06	*w* = 1/[σ^2^(*F*_o_^2^) + (0.0633*P*)^2^] where *P* = (*F*_o_^2^ + 2*F*_c_^2^)/3
1228 reflections	(Δ/σ)_max_ < 0.001
169 parameters	Δρ_max_ = 0.19 e Å^−^^3^
0 restraints	Δρ_min_ = −0.21 e Å^−^^3^

### Special details

**Table:**

Geometry. All e.s.d.'s (except the e.s.d. in the dihedral angle between two l.s. planes) are estimated using the full covariance matrix. The cell e.s.d.'s are taken into account individually in the estimation of e.s.d.'s in distances, angles and torsion angles; correlations between e.s.d.'s in cell parameters are only used when they are defined by crystal symmetry. An approximate (isotropic) treatment of cell e.s.d.'s is used for estimating e.s.d.'s involving l.s. planes.
Refinement. Refinement of *F*^2^ against ALL reflections. The weighted *R*-factor *wR* and goodness of fit *S* are based on *F*^2^, conventional *R*-factors *R* are based on *F*, with *F* set to zero for negative *F*^2^. The threshold expression of *F*^2^ > σ(*F*^2^) is used only for calculating *R*-factors(gt) *etc*. and is not relevant to the choice of reflections for refinement. *R*-factors based on *F*^2^ are statistically about twice as large as those based on *F*, and *R*- factors based on ALL data will be even larger.

### Fractional atomic coordinates and isotropic or equivalent isotropic displacement parameters (Å^2^)

**Table:**

	*x*	*y*	*z*	*U*_iso_*/*U*_eq_	Occ. (<1)
C1	0.5852 (2)	1.1330 (5)	0.13062 (12)	0.0203 (7)	
O1	0.57188 (16)	1.3797 (3)	0.11721 (8)	0.0291 (6)	
C2	0.5376 (2)	1.0250 (4)	0.18985 (11)	0.0177 (7)	
C7	0.4271 (2)	1.1328 (5)	0.20975 (13)	0.0229 (7)	
H7	0.3828	1.2677	0.1850	0.027*	0.856 (4)
F1	0.71053 (15)	0.7317 (3)	0.21278 (8)	0.0296 (7)	0.856 (4)
C3	0.5996 (2)	0.8335 (5)	0.22854 (13)	0.0242 (7)	
H3	0.6757	0.7577	0.2162	0.029*	0.144 (4)
F1'	0.3678 (10)	1.321 (2)	0.1771 (6)	0.046 (4)	0.144 (4)
C4	0.5566 (3)	0.7481 (5)	0.28378 (13)	0.0302 (7)	
H4	0.6022	0.6179	0.3093	0.036*	
C5	0.4450 (3)	0.8563 (5)	0.30152 (13)	0.0284 (7)	
H5	0.4126	0.7979	0.3393	0.034*	
C6	0.3806 (2)	1.0486 (5)	0.26455 (12)	0.0270 (7)	
H6	0.3043	1.1228	0.2769	0.032*	
N1	0.64105 (18)	0.9476 (5)	0.09499 (10)	0.0197 (6)	
HN1	0.639 (2)	0.781 (5)	0.1077 (12)	0.028 (8)*	
C8	0.6910 (2)	1.0053 (4)	0.03731 (11)	0.0173 (7)	
C9	0.8010 (2)	0.8696 (4)	0.02298 (12)	0.0186 (7)	
C14	0.8660 (2)	0.6685 (5)	0.06812 (12)	0.0243 (7)	
H14A	0.8801	0.7574	0.1088	0.036*	
H14B	0.9463	0.6140	0.0528	0.036*	
H14C	0.8138	0.5037	0.0720	0.036*	
C10	0.8481 (2)	0.9264 (5)	−0.03388 (12)	0.0242 (7)	
H10	0.9220	0.8353	−0.0446	0.029*	
C11	0.7908 (2)	1.1116 (5)	−0.07566 (12)	0.0264 (7)	
H11	0.8257	1.1480	−0.1141	0.032*	
C12	0.6822 (2)	1.2436 (5)	−0.06095 (13)	0.0235 (7)	
H12	0.6421	1.3704	−0.0894	0.028*	
C13	0.6326 (2)	1.1905 (5)	−0.00504 (12)	0.0205 (7)	
H13	0.5579	1.2807	0.0049	0.025*	

### Atomic displacement parameters (Å^2^)

**Table:**

	*U*^11^	*U*^22^	*U*^33^	*U*^12^	*U*^13^	*U*^23^
C1	0.0195 (15)	0.0157 (16)	0.0256 (18)	−0.0019 (12)	0.0003 (13)	0.0007 (13)
O1	0.0434 (12)	0.0128 (11)	0.0326 (12)	0.0013 (8)	0.0142 (9)	0.0032 (8)
C2	0.0229 (15)	0.0104 (13)	0.0197 (16)	−0.0033 (12)	0.0005 (13)	−0.0007 (11)
C7	0.0246 (16)	0.0188 (15)	0.0253 (18)	−0.0012 (13)	0.0015 (14)	−0.0003 (12)
F1	0.0306 (11)	0.0303 (11)	0.0279 (12)	0.0111 (9)	0.0007 (9)	0.0041 (8)
C3	0.0245 (17)	0.0213 (16)	0.0268 (19)	−0.0004 (12)	0.0028 (14)	−0.0024 (12)
F1'	0.050 (8)	0.044 (8)	0.046 (9)	0.018 (6)	0.011 (6)	0.015 (6)
C4	0.0394 (18)	0.0271 (16)	0.0236 (19)	−0.0024 (14)	−0.0015 (15)	0.0038 (13)
C5	0.0409 (18)	0.0276 (16)	0.0170 (17)	−0.0117 (14)	0.0051 (14)	0.0006 (12)
C6	0.0278 (16)	0.0292 (16)	0.0246 (18)	−0.0018 (13)	0.0060 (13)	−0.0021 (13)
N1	0.0258 (13)	0.0101 (13)	0.0239 (15)	0.0023 (11)	0.0060 (11)	0.0022 (11)
C8	0.0203 (15)	0.0113 (14)	0.0206 (17)	−0.0051 (11)	0.0028 (13)	−0.0002 (11)
C9	0.0212 (15)	0.0125 (14)	0.0216 (17)	−0.0031 (11)	−0.0008 (13)	−0.0027 (11)
C14	0.0244 (15)	0.0227 (15)	0.0259 (18)	0.0031 (12)	0.0022 (13)	−0.0016 (12)
C10	0.0234 (15)	0.0241 (15)	0.0255 (18)	0.0002 (13)	0.0040 (13)	−0.0049 (13)
C11	0.0305 (17)	0.0291 (16)	0.0199 (17)	−0.0047 (13)	0.0043 (14)	−0.0017 (13)
C12	0.0295 (16)	0.0188 (15)	0.0216 (18)	−0.0042 (13)	−0.0033 (13)	0.0034 (12)
C13	0.0173 (14)	0.0163 (14)	0.0278 (19)	−0.0020 (11)	−0.0007 (13)	−0.0003 (12)

### Geometric parameters (Å, °)

**Table:**

C1—O1	1.231 (3)	N1—C8	1.416 (3)
C1—N1	1.347 (3)	N1—HN1	0.85 (3)
C1—C2	1.501 (3)	C8—C13	1.397 (3)
C2—C3	1.386 (3)	C8—C9	1.404 (3)
C2—C7	1.390 (3)	C9—C10	1.385 (4)
C7—F1'	1.292 (10)	C9—C14	1.512 (3)
C7—C6	1.375 (4)	C14—H14A	0.9800
C7—H7	0.9500	C14—H14B	0.9800
F1—C3	1.354 (3)	C14—H14C	0.9800
C3—C4	1.370 (4)	C10—C11	1.384 (3)
C3—H3	0.9500	C10—H10	0.9500
C4—C5	1.385 (4)	C11—C12	1.385 (4)
C4—H4	0.9500	C11—H11	0.9500
C5—C6	1.379 (4)	C12—C13	1.375 (4)
C5—H5	0.9500	C12—H12	0.9500
C6—H6	0.9500	C13—H13	0.9500
			
O1—C1—N1	123.9 (2)	C13—C8—N1	121.4 (2)
O1—C1—C2	119.7 (2)	C9—C8—N1	118.4 (2)
N1—C1—C2	116.5 (2)	C13—C8—C14^i^	76.61 (13)
C3—C2—C7	116.7 (2)	C9—C8—C14^i^	90.97 (14)
C3—C2—C1	124.5 (2)	N1—C8—C14^i^	103.13 (14)
C7—C2—C1	118.6 (2)	C10—C9—C8	117.7 (2)
F1'—C7—C6	118.8 (6)	C10—C9—C14	121.2 (2)
F1'—C7—C2	119.7 (6)	C8—C9—C14	121.1 (2)
C6—C7—C2	121.4 (2)	C9—C14—H14A	109.5
C6—C7—H7	119.3	C9—C14—H14B	109.5
C2—C7—H7	119.3	H14A—C14—H14B	109.5
F1—C3—C4	117.6 (2)	C9—C14—H14C	109.5
F1—C3—C2	119.1 (2)	H14A—C14—H14C	109.5
C4—C3—C2	123.2 (2)	H14B—C14—H14C	109.5
C4—C3—H3	118.4	C11—C10—C9	122.1 (2)
C2—C3—H3	118.4	C11—C10—H10	118.9
C3—C4—C5	118.4 (3)	C9—C10—H10	118.9
C3—C4—H4	120.8	C10—C11—C12	119.5 (3)
C5—C4—H4	120.8	C10—C11—H11	120.2
C6—C5—C4	120.2 (3)	C12—C11—H11	120.2
C6—C5—H5	119.9	C13—C12—C11	119.8 (2)
C4—C5—H5	119.9	C13—C12—H12	120.1
C5—C6—C7	120.0 (3)	C11—C12—H12	120.1
C5—C6—H6	120.0	C12—C13—C8	120.6 (2)
C7—C6—H6	120.0	C12—C13—C14^i^	88.08 (15)
C1—N1—C8	125.5 (2)	C8—C13—C14^i^	81.74 (14)
C1—N1—HN1	115.0 (18)	C12—C13—H13	119.7
C8—N1—HN1	119.2 (18)	C8—C13—H13	119.7
C13—C8—C9	120.2 (2)	C14^i^—C13—H13	100.3
			
O1—C1—C2—C3	−140.1 (3)	C1—N1—C8—C13	−37.4 (3)
N1—C1—C2—C3	39.8 (3)	C1—N1—C8—C9	143.2 (2)
O1—C1—C2—C7	36.3 (3)	C1—N1—C8—C14^i^	44.8 (3)
N1—C1—C2—C7	−143.8 (2)	C13—C8—C9—C10	0.0 (3)
C3—C2—C7—F1'	176.9 (6)	N1—C8—C9—C10	179.4 (2)
C1—C2—C7—F1'	0.3 (7)	C14^i^—C8—C9—C10	−75.1 (2)
C3—C2—C7—C6	−1.4 (3)	C13—C8—C9—C14	179.9 (2)
C1—C2—C7—C6	−178.0 (2)	N1—C8—C9—C14	−0.6 (3)
C7—C2—C3—F1	−176.9 (2)	C14^i^—C8—C9—C14	104.9 (2)
C1—C2—C3—F1	−0.5 (3)	C8—C9—C10—C11	0.6 (3)
C7—C2—C3—C4	0.5 (4)	C14—C9—C10—C11	−179.4 (2)
C1—C2—C3—C4	176.9 (2)	C9—C10—C11—C12	−0.7 (4)
F1—C3—C4—C5	178.2 (2)	C10—C11—C12—C13	0.3 (4)
C2—C3—C4—C5	0.7 (4)	C11—C12—C13—C8	0.3 (3)
C3—C4—C5—C6	−1.0 (4)	C11—C12—C13—C14^i^	79.5 (2)
C4—C5—C6—C7	0.2 (4)	C9—C8—C13—C12	−0.4 (3)
F1'—C7—C6—C5	−177.3 (6)	N1—C8—C13—C12	−179.8 (2)
C2—C7—C6—C5	1.0 (4)	C14^i^—C8—C13—C12	82.8 (2)
O1—C1—N1—C8	−0.8 (4)	C9—C8—C13—C14^i^	−83.2 (2)
C2—C1—N1—C8	179.30 (19)	N1—C8—C13—C14^i^	97.4 (2)

Symmetry codes: (i) *x*, *y*+1, *z*.

### Hydrogen-bond geometry (Å, °)

**Table:**

*D*—H···*A*	*D*—H	H···*A*	*D*···*A*	*D*—H···*A*
N1—HN1···O1^ii^	0.85 (3)	2.08 (3)	2.887 (3)	158 (2)
C6—H6···F1'^iii^	0.95	2.60	3.219 (11)	123
C12—H12···O1^iv^	0.95	2.63	3.434 (3)	143
C12—H12···F1'^iv^	0.95	2.40	3.287 (11)	154
C14—H14C···Cg1^ii^	0.98	2.98	3.702 (3)	131

Symmetry codes: (ii) *x*, *y*−1, *z*; (iii) −*x*+1/2, *y*−1/2, −*z*+1/2; (iv) −*x*+1, −*y*+3, −*z*.
